# Facilitators and Barriers to Post-partum Diabetes Screening Among Mothers With a History of Gestational Diabetes Mellitus–A Qualitative Study From Singapore

**DOI:** 10.3389/fendo.2020.00602

**Published:** 2020-08-28

**Authors:** Sharon Hanna Sunny, Rahul Malhotra, Seng Bin Ang, C. S. Daniel Lim, Y. S. Andrew Tan, Y. M. Benjy Soh, X. Y. Cassandra Ho, Martyn Gostelow, L. P. Marianne Tsang, S. H. Smily Lock, Suat Yee Kwek, Y. T. Jana Lim, Kayshini Vijakumar, Ngiap Chuan Tan

**Affiliations:** ^1^Duke-NUS Medical School, Singapore, Singapore; ^2^Family Medicine Service, Kandang-Kerbau Women and Children Hospital, Singapore, Singapore; ^3^SingHealth-Duke NUS Family Medicine Academic Clinical Programme, Singapore, Singapore; ^4^SingHealth Polyclinics, Singapore, Singapore

**Keywords:** gestational diabetes mellitus, post-partum diabetes screening, facilitators, barriers, socio-ecological model

## Abstract

**Introduction:** Gestational Diabetes Mellitus (GDM) affects one in six births worldwide. Mothers with GDM have an increased risk of developing post-partum Type-2 Diabetes Mellitus (T2DM). However, their uptake of post-partum diabetes screening is suboptimal, including those in Singapore. Literature reports that the patient-doctor relationship, mothers' concerns about diabetes, and family-related practicalities are key factors influencing the uptake of such screening. However, we postulate additional factors related to local society, healthcare system, and policies in influencing post-partum diabetes screening among mothers with GDM.

**Aim:** The qualitative research study aimed to explore the facilitators and barriers to post-partum diabetes screening among mothers with GDM in an Asian community.

**Methods:** In-depth interviews were carried out on mothers with GDM at a public primary care clinic in Singapore. Mothers were recruited from those who brought their child for vaccination appointments and their informed consent was obtained. Both mothers who completed post-partum diabetes screening within 12 weeks after childbirth and those who did not were purposively recruited. The social ecological model (SEM) provides the theoretical framework to identify facilitators and barriers at the individual, interpersonal, organizational, and policy levels.

**Results:** Twenty multi-ethnic Asian mothers with GDM were interviewed. At the individual and interpersonal level, self-perceived risk of developing T2DM, understanding the need for screening and the benefits of early diagnosis, availability of confinement nanny in Chinese family, alternate caregivers, emotional, and peer support facilitated post-partum diabetes screening. Barriers included fear of the diagnosis and its consequences, preference for personal attention and care to child, failure to find trusted caregiver, competing priorities, and unpleasant experiences with the oral glucose tolerance test. At the organizational and public policy level, bundling of scheduled appointments, and standardization of procedure eased screening but uptake was hindered by inconvenient testing locations, variable post-partum care practices and advice in the recommendations for diabetes screening.

**Conclusion:** Based on the SEM, facilitators and barriers towards post-partum diabetes screening exist at multiple levels, with some contextualized to local factors. Interventions to improve its uptake should be multi-pronged, targeting not only at personal but also familial, health system, and policy factors to ensure higher level of success.

## Introduction

According to the International Diabetes Federation, up to 20.4 million live births in 2019 were complicated by hyperglycaemia (1). Gestational Diabetes Mellitus (GDM), defined as any extent of hyperglycaemia first identified during pregnancy (2), particularly affects the South-East Asian region which has the highest prevalence of GDM in the world (3). The prevalence of GDM in Singapore, at the centre of South-East Asia, is estimated to be 18.9% (4). This is of concern as mothers with GDM are not only more likely to have hyperglycaemia in subsequent pregnancies (5) but also have a 7-fold increased risk of developing Type 2 Diabetes Mellitus (T2DM) (6). A systematic review and meta-analysis found that this risk was the highest within 3 to 6 years after the affected pregnancy (7), thus necessitating timely and appropriate screening regimens to mitigate the risk.

For early identification and management of T2DM, international and local guidelines recommend that mothers with GDM undergo screening for persistent dysglycemia at 6 to 12 weeks post-partum, with the recommended 75 g 2-h oral glucose tolerance test (OGTT) (8, 9). Whilst up to 18.2% of mothers screened are diagnosed with dysglycemia (10), the uptake of post-partum diabetes screening within the recommended window is suboptimal and varies widely across populations. A study conducted in England identified the uptake rate to be 17% (11), as compared to 81.9% in a Malaysian hospital (12). Unpublished data from a tertiary care institution in Singapore shows that just over half (54%) of mothers with GDM underwent post-partum diabetes screening within the recommended time frame. This calls for the identification of facilitators and barriers to postpartum diabetes screening among mothers with GDM.

The reasons for suboptimal uptake of post-partum diabetes screening have been assessed in Western populations (13). A systematic review in 2019, based on 16 qualitative research studies, detailed four major themes. These were broadly classified according to the health-care system and personal factors such as the mother's relationship with her physician; experience of the OGTT; mother's perceived risk of T2DM and family-related complexities (14). Singaporean mothers with GDM probably encounter similar facilitators and barriers. However, we postulate that the structure of the local health-care system and societal practices may also contribute to the suboptimal uptake of post-partum diabetes screening. In Singapore, the health-system is two-tiered, consisting of public and private health care institutions (15). Differences in demography, family structure, support, education status, social interactions, cultural, and religious background of the multi-ethnic Asian mothers may also affect the screening uptake in Singapore (16).

A framework to provide clarity to the potential interplay of personal, familial, and societal factors (17) would be ideal to understand the complex issues affecting the screening uptake. The Socio-Ecological Model (SEM) seems to be a suitable framework as it posits the role of individual, interpersonal, organisational, and public policy factors in determining health behaviour (18). It has been used widely in the study of health promotion (19). For example, the United States Department of Health and Human Services utilized the four domains of the SEM in the creation of its national objectives in 2020, thereby acknowledging its comprehensive purview in understanding the factors affecting health behaviour (20).

This qualitative research study aimed to explore the facilitators and barriers to post-partum diabetes screening among mothers with GDM in Singapore. These findings can be used to guide the development of multi-pronged strategies to improve the uptake of post-partum diabetes screening.

## Materials and Methods

### Study Design

We used in-depth, semi-structured interviews to identify the facilitators and barriers to post-partum diabetes screening among mothers with GDM in Singapore. The data collected was analyzed for emerging themes, which were subsequently presented using the SEM.

### Site

The study was conducted at Punggol Polyclinic, a public primary care clinic that serves an estate populated with young families in northeast Singapore. The clinic manages at least 900 patient attendances daily, with a special focus on women's and children's health.

### Period of Study

The study was conducted between October 2019 and January 2020.

### Study Population

The target participants were Singapore citizen or permanent resident mothers with a self-reported diagnosis of GDM in their most recent pregnancy, and with a child aged 3 to 6 months at the time of the interview. A lower limit of 3 months was stipulated to ensure that mothers had already attended or not attended their post-partum diabetes screening within the recommended time frame, and an upper limit of 6 months to reduce recall bias. They also had to be English-literate as the interviews were conducted in English, one of the official languages of Singapore. Those with a pre-existing diagnosis of Type 1 and Type 2 Diabetes Mellitus were excluded.

### Recruitment

**Figure 1:**
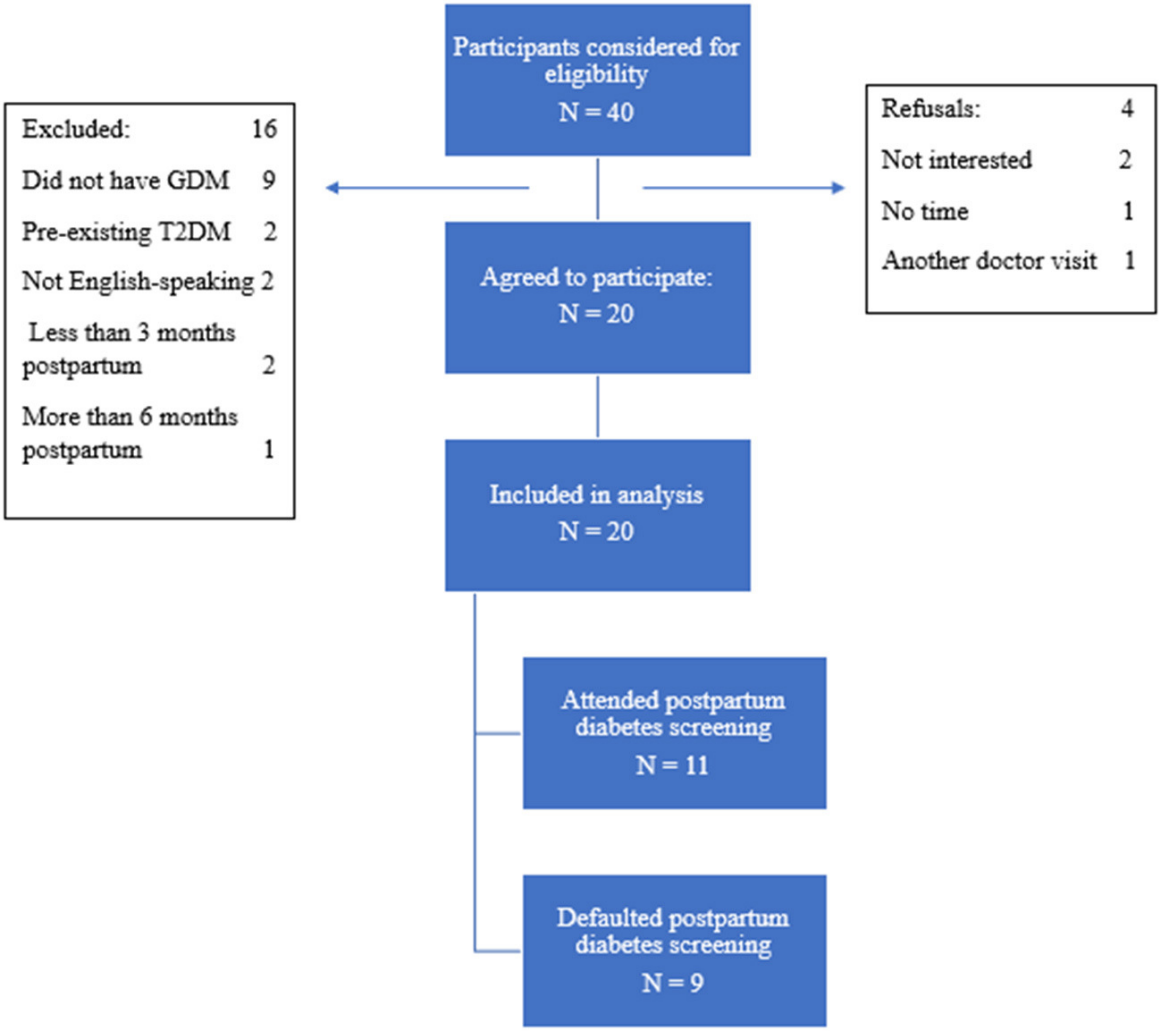
Recruitment process of patients.

Potential participants brought their child to the study site for their routine childhood vaccination and developmental assessment. Mothers with GDM were directed by the nurses providing these services to the lead investigator, SHS on a consecutive case-encounter basis. SHS provided eligible participants with study-related information and clarified their doubts before obtaining their written informed consent. The recruitment process is described in Figure 1. Purposive sampling was intended to include women of different ethnic groups to identify specific cultural and societal practice related to ethnicity.

### Conceptual Framework

The SEM was selected as the conceptual framework to identify the facilitators and barriers across the personal, interpersonal, organizational, and public policy domains. Resulting themes were subsequently organized and presented according to the SEM.

### Interviews

A total of 20 mothers were recruited. They completed a demographic data questionnaire before commencing the one-to-one interview, which lasted between 20 to 30 min. Mothers were reimbursed with grocery store vouchers of SGD20 value for their time.

### Coding

The interviews were audio-recorded and transcribed verbatim. Each interview was independently coded by two investigators and the coding was subsequently discussed with other investigators. The first nine interviews were reviewed to form a coding frame to guide the analysis of the remaining 11 interviews.

## Results

Table 1 presents the demographic characteristics of the 20 participants. Ten of them delivered in public hospitals. All ten were reminded to return for post-partum diabetes screening, of whom eight subsequently went for their post-partum diabetes screening. The remaining ten mothers delivered in private hospitals, three of whom were reminded and returned for screening.

**Table 1:** Demographic characteristics of study participants (*n* = 20).

**Characteristics**	***N* (%)**
**Age (years)**	
26–30	3 (15%)
31–35	15 (80%)
36–40	2 (10%)
**Ethnicity: (Percentage of ethnic group in study and national population)**	
Chinese	13 (65%/76%)
Malay	5 (25%/15%)
Indian	1 (5%/7.5%)
Other	1 (5%/1.5%)
Primiparous	9 (45%)
Diagnosis of GDM in previous pregnancies	4 (20%)
Family/friends with GDM	11 (55%)
Family/friends with DM	11 (55%)
**Highest educational level**	
Secondary (O, N levels)	4 (20%)
ITE	4 (20%)
Polytechnic	3 (15%)
University	9 (45%)
**Housing type**	
1 to 3-room HDB flat	2 (10%)
4-room or bigger HDB flat	18 (90%)
**Post-partum diabetes screening**	
Attended	11
Defaulted	9
**Site of antenatal/post-partum care**	
Public healthcare institution	10
Private healthcare institution	10

*GDM, Gestational Diabetes Mellitus; DM, Diabetes Mellitus; ITE, Institute of Technical Education; HDB, Housing Development Board*.

**Figure 2:**
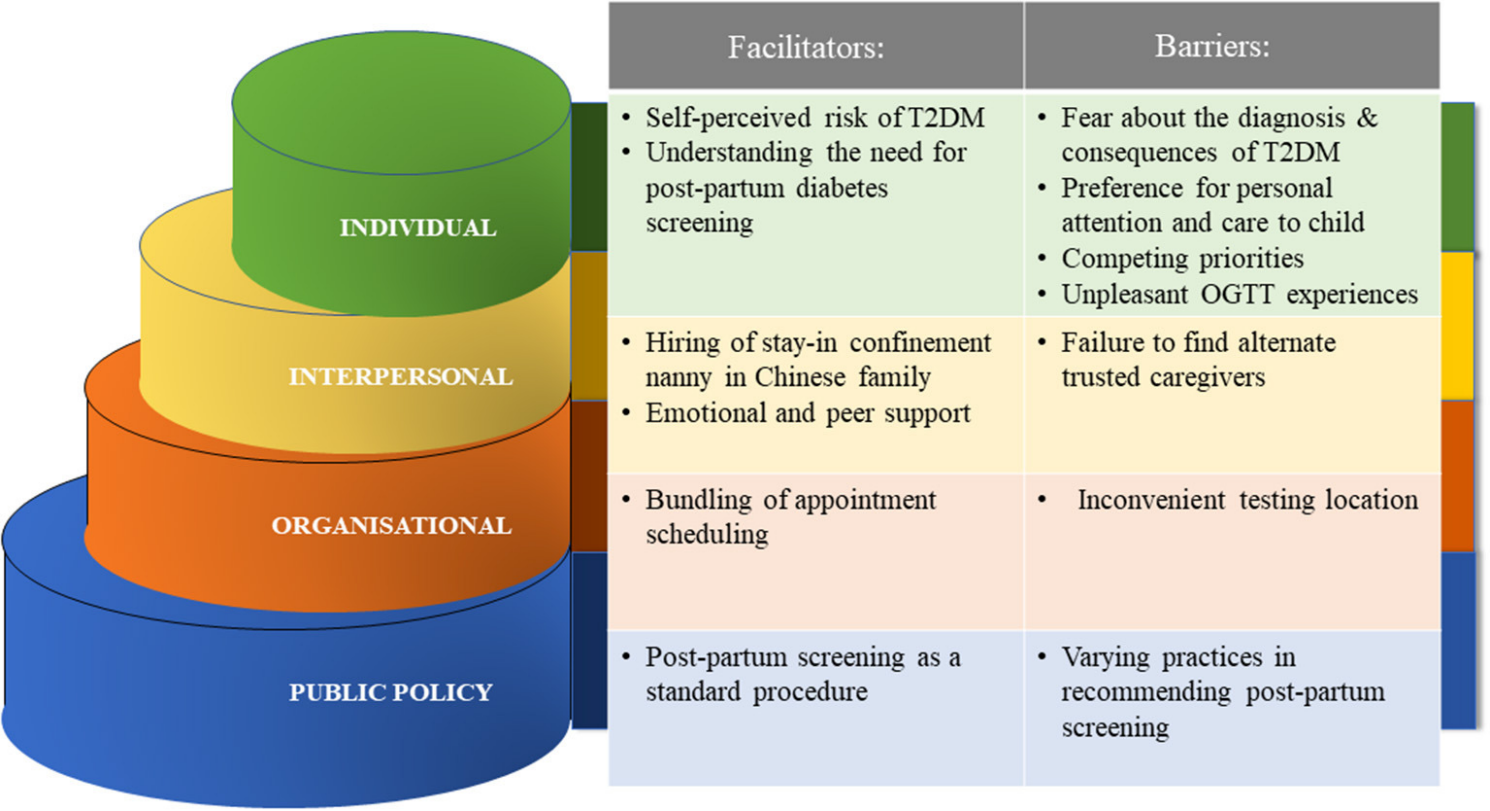
Facilitators and barriers to post-partum diabetes screening based on the Socio-Ecological Model.

The facilitators and barriers were organized according to the domains of the SEM (Figure 2). Verbatim quotes from the participants were selected to illustrate the themes.

### Individual-Level

#### Facilitators at the Individual-Level

##### Self-perceived risk of developing T2DM

Mothers who were aware of their increased risk of developing T2DM were more likely to return for screening. They attained this knowledge from various sources of information, such as online readings, doctor recommendations, and family members who had T2DM.

“*But she [mother's obstetrician] did mention that I may have a risk, since I had GDM when I was pregnant … because of my size, um that's quite unlikely for a pregnant lady to have GDM. So usually, it's uh, people who have bigger sizes. So, … I may have (been) a pre-diabetic.”* P1, Chinese, attended screening at private hospital.

“*Because my age is 34 […] I know my parents, genetics…That's why I am worried, that's why I want to go. Genetically maybe it will continue… Later pregnancy…it [GDM] will come.”* P3, Indian, attended screening at a public hospital.

##### Understanding the need for post-partum diabetes screening

Some mothers undertook post-partum diabetes screening because they understood the rationale for the test. They were aware that the diagnosis of diabetes would impact their lifestyle habits and place them at higher risk of complications in subsequent pregnancies. These mothers recognized the need for behavior change after the affirmative results from the screening tests.

“*No, I just have to find out, because if I had known earlier, then I would just have to take note, ok, what I can do from there onwards. If not, I'll never know and then I'll splurge on all the stuff that I have been wanting to eat.”* P1, Chinese, attended screening at private hospital.

#### Barriers at the Individual Level

##### Fear about the diagnosis and consequences of T2DM

Some mothers did not go for screening as they were reluctant to find out if they had diabetes for fear that the diagnosis might disadvantage them. For instance, one mother expressed concern that this diagnosis would affect her and her child's insurance premiums.

“*this sickness will follow you throughout your life … people will always ask, like even the doctor or like the insurer, they ask you this kind of question, do you ever have like diabetes or anything”* P12, Chinese, defaulted her screening at a private hospital.

##### Preference for personal attention and care to child

Some mothers preferred personal attention to their infant and other older children, and felt uneasy for an alternative caregiver to look after them.

“*Yeah but it does pose a challenge, you know, right after that one month, where we are still … very new with the baby. You need a lot of attention and we are not sure what to do when you are away from the baby, whether another person will be able to manage. So yeah there's this concern. Maybe this is the challenge for other women.”* P16, Chinese, attended screening at a public hospital.

##### Competing priorities

Women prioritized the comfort and needs of their children over their personal health.

“*because of the fasting, they will make my appointment, the first thing in the morning… around 8 plus. And she (child) usually isn't up by then. Yeah, so it will be disruptive to her sleep, if I have to stay there for 2 h. The journey back and forth will be a bit tough.”* P20, Chinese, attended screening at a private hospital.

“*got appointment [referring to OGTT at hospital], and then got um therapy at school [older son's speech therapy]”* P7, Malay, defaulted screening at a private hospital.

##### Unpleasant OGTT experience

Many of the mothers were averse to the 75 g 2-point OGTT test, which they experienced during their pregnancies. This sentiment was shared widely both by those who had the post-partum screening and those who defaulted. The deterrents included the long duration of the test, the unpalatable taste of the glucose syrup, its perceived inaccuracy and the repeated venipunctures required.

“*It's a bit uh… long. And the water [glucose syrup] is very sweet. It's like not, not very fair, because we drink the water, it's so sweet and they don't let us drink water or walk around. It's unfair. Because we don't do that in our daily life. We will move around and we drink water. So … I think the test might not be so accurate on that part.”* P4, Chinese, defaulted screening at private hospital.

“*I will be able to but … I don't feel like* going […] *Uhhh! The syrup is so disgusting, it's like ummm (makes a grimace), and after drinking, you feel like giddiness, you cannot stand, and walk around. You have to, like, sit down there and rest.”* P12, Chinese, defaulted screening at private hospital.

“*Wa! It's like F& N orange [soft drink brand in Singapore], but very, very, very, very, very sweet one! Sweet until you… [mimes gagging] vomit!”* P8, Chinese, had screening at a private hospital.

### Interpersonal-Level

#### Facilitators at the Interpersonal-Level

##### Hiring of stay-in confinement nanny in Chinese family

The hiring of stay-in confinement nanny is more common amongst the Chinese women during the immediate 1 month after their delivery. One mother was able to attend the screening after leaving her child with her stay-in confinement nanny.

“*my confinement lady took care of [baby] so I'm able to come out and do the test and go for my gynae review”* P1, Chinese, attended screening at a private hospital.

##### Emotional and peer support

A number of mothers favored a support network that encouraged them to return for post-partum screening. They described the emotional support from spouses, friends, online support groups comprising mothers with similar experiences of GDM, and healthcare professionals. Such support strengthened their decision to undertake the screening test.

“*my husband usually helps me…I plan to have the right time when my husband can actually take leave. When the kids are on holidays, or they are not schooling… that will be the best time for extra help. My husband (is) at home, cos he can take care, looks after the 2 small ones.”* P9, Malay, attended screening at public hospital.

“*Absolutely, they will encourage me! Go and check, every time go and check the sugar levels. … the parents and husband, they are very encouraging.”* P3, Indian, attended screening at a public hospital.

“*So, going through those conversations [in online ‘mummy chat groups’] helped a lot*. P1, Chinese, attended screening at private hospital.

#### Barriers at the Interpersonal-Level

##### Failure to find alternate trusted caregivers

Not every mother had ready access to alternative caregivers. They preferred immediate family member such as their spouse, parents or in-laws to look after the child in their absence. This was a common reason for mothers to default their post-partum screening test. One mother recounted that her husband was the sole breadwinner and was not available to take over caregiving for their child.

“*because my husband working night shift then he [didn't] sleep…*” P7, Malay, defaulted screening at a public hospital.

### Organizational-Level

#### Facilitators at the Organizational-Level

##### Bundling of scheduled appointments

A number of mothers preferred bundling their screening test appointments with other post-partum investigations, such as Pap smears, for convenience.

“*I also thought it was good, at the three-month mark, to see my gynae, for other reasons, for him to just check. I think he wanted to do a Pap smear. So, uh, just doing it all together made it convenient.”* P20, Chinese, attended screening at private hospital.

“*The main thing [that caused the mother to default post-partum screening in her previous pregnancy] is the busy schedule. [For her latest pregnancy] because I have the Pap smear there too…same day … so that's why I think, just one day off.”* P4, Chinese, defaulted screening at private hospital.

#### Barriers at the Organizational-Level

##### Inconvenient testing locations

A few mothers expressed reluctance to return to their antenatal care providers for screening due to the long distance from their residences to their obstetricians' clinics. A few also preferred their screening to be at primary care clinics (polyclinics), as compared to hospitals, due to their perceived shorter wait times.

“*I don't really have all the time to go all the way to KK [tertiary public healthcare institution]* P10, other ethnic group, defaulted screening at public hospital.

“*…instead of going to KK [tertiary public healthcare institution], maybe polyclinics can do it also? … cos they [tertiary public healthcare institution] deal with a lot of people, the waiting time is quite long”* P13, Malay, attended screening at public hospital.

### Public Policy-Level

#### Facilitators at the Public Policy-Level

##### Post-partum screening as a standard procedure

Mothers' perception of obligatory screening for T2DM was key to their uptake of the test.

“*I thought it's like mandatory?”* P16, Chinese, attended screening at a public hospital.

“*No, it's like a routine, so just took it.”* P1, Chinese, attended screening at a private hospital.

#### Barriers at the Public Policy-Level

##### Varying practices in recommending post-partum screening

Some mothers reported a lack of advice and recommendations for T2DM screening from their antenatal care providers despite their diagnosis of GDM during their pregnancies. In particular, one mother recounted that she had to request for screening personally, as it was not offered as part of her pregnancy care “package”.

“*…nobody asked me for a check, so I didn't bother to follow-up.”* P6, Chinese, defaulted screening at private hospital.

“*But the gynae said don't need…then no need […]! I just trust him.”* P12, Chinese, defaulted screening at private hospital.

The test of choice for post-partum screening also varied amongst providers, with some offering random blood glucose tests or in-office finger-prick tests instead of an OGTT. Few also revealed that their diagnoses of GDM were dismissed or downplayed when additional random blood glucose tests during pregnancy were normal.

“*So maybe it was because of the impromptu blood test that was done without fasting so at like random timing and the value is very good […]. So I think he was not that worried about my GD [referring to GDM]. Maybe it's just borderline case.”* P14, Chinese, defaulted screening at private hospital.

## Discussion

This study elucidated facilitators and barriers for post-partum diabetes screening among mothers with GDM across multiple domains. While the results largely echo those identified by systematic reviews, new themes have been identified at the interpersonal and public policy levels which are distinctive to Asian mothers in Singapore.

At the individual level, mothers who had greater knowledge about the risks of GDM and T2DM were more likely to take up the screening. Therefore, healthcare professionals should educate mothers about GDM, T2DM, and the importance of post-partum diabetes screening actively, even during antenatal visits (21). A systematic review revealed the short-term relationship between mother and their antenatal care provider ended soon after their delivery (13). Hence health messages were not reinforced to the mothers in the post-partum period by any healthcare professionals. The gap in care can be addressed with proper handover of care to primary care physicians to continue their health monitoring. Mothers who were reassured that GDM was only a “mild condition of pregnancy” were also not as motivated to return for screening (22). Primary care physicians have a role to play in correcting some of these misconceptions during their postnatal visits.

For mothers who are undecided on their post-partum diabetes screening, or have not been adequately counselled on their risks after delivery, a Patient Decision Aid (PDA) can encourage shared-decision making between them and their physician (23). A local pilot study at a public hospital found that mothers perceived that they had received adequate “material,” “emotional,” and “comparison” support. However, they claimed inadequacy of “informational” support despite the abundance of informational pamphlets and brochures which were available to them (24). The investigators are developing a PDA targeting women with GDM on postnatal diabetes screening which may potentially overcome this lack of “informational support.” PDA provides a convenient platform to trigger discussion on postnatal diabetes screening if it is readily accessible to the at-risk women at any clinical practice. Aside from presenting balanced perspectives of the screening test, including its benefits and inconvenience, the PDA will also offer tips to address common barriers such as availability of caregivers. Such PDA can be implemented in public and private healthcare practices to reach out to more women with GDM. It will be assessed for its effectiveness to increase uptake of the screening in the next phase of this project.

The use of alternative screening tests which may be more convenient or pleasant than the OGTT should be explored. The latest National Institute of Care and Excellence guidelines from the UK for post-partum diabetes screening suggest the use of a fasting plasma glucose test at 6 to 13 weeks after delivery. If a fasting glucose test has not been performed by 13 weeks, offer a fasting plasma glucose test, or an HbA1c test if a fasting plasma glucose test is not possible, after 13 weeks (25). Women will not be required to consume the glucose drink, which most Asian women in this study found distasteful and unpleasant. However, other studies reported the HbA1c to have a low sensitivity of only 14.3% in diagnosing T2DM in the post-partum population when compared to the OGTT (26). Hence the validity of diabetes screening tests other than the OGTT for the diagnosis of T2DM in mothers with GDM remains unclear.

At the interpersonal level, most women in our study reported that their child was cared for by their family members (mother, mother-in-law or spouse) while they undertook the post-partum diabetes screening. Asian women appeared to prefer personal attention and care of their child; otherwise they will entrust their child to close family members during their absence. The stay-in confinement nanny is a convenient and immediate caregiver to assist the mother. Almost one third (31%) of women of Chinese ethnic group hired such confinement assistants in a local study by Fok et al. which is less common in other ethnic groups such as the Malay (13.5%) and Indian (9.4%). (27) The confinement period usually lasts between 30 and 45 days. The women's mothers and mother-in-laws are the other major groups of care providers during the confinement period, ranging from 59.4% in Chinese to 71.5% in Malay and 83.3% in Indians. They are also trusted caregivers to take care of the child, if the screening test can be scheduled at 6-week post-partum, which is at the end of confinement period (27).

In addition, most women did not wish to bring their child to the clinic. Fok et al. also reported that Chinese mothers (83.7%) were least likely to bring their child outside the home compared to Indians (79.9%) and Malays (66.1%) (27). If possible, mothers can consider seeking help in looking after their child from their parents, in-laws, spouses, siblings, or even trusted neighbors while they attend their postnatal physician visits (28). Public and social policies such as paternity leave for fathers (29) or encouraging young families to stay near to their parents through housing incentives (30) may also help to address this barrier.

In addition, healthcare institutions can consider offering on-site childcare services or provide play areas for older children, as suggested by many mothers. This proposal aligns with the recommendations suggested by Dennison et al. in their systematic review (14), and will apply not just for postpartum visits but will facilitate mothers seeking medical attention.

At the organizational level, the bundling of the post-partum diabetes screening with other post-partum review can optimize the time and utility of each visit. Mothers should be made aware of such options early during their antenatal visits via clear, uniform instructions by both their obstetricians and primary care physicians. As these test can be planned weeks in advance, the option of scheduling a mother's post-partum diabetes screening with other appointments at suitable locations should be offered routinely by the institution (14).

Clinical practice guidelines are available to recommend the routine screening for T2DM in mothers with GDM (31). However, the variable adherence by healthcare providers to such guidelines, especially with a local two-tiered healthcare system, poses challenges to their implementation. Differences were noted from the recommendations by public and private healthcare providers, which could be due to lack of effective policies to ensure consistent adherence to the guidelines.

The inconsistent handover from obstetricians to other healthcare providers after delivery further compounds the problem. Local mothers can now access their National Electronic Health Records (NEHR) remotely using computers or smart mobile phones. However, while it is implemented across all public healthcare institutions, adoption by private healthcare providers is low. Only 27% of local private healthcare institutions have access to NEHR and a mere 3% of them contribute data (32). Therefore, details about a mother's glycemic control during and after pregnancy may not be readily available should she attend private primary care clinics or obstetricians. While we await a unified nationwide electronic health record, healthcare policy-makers may leverage on existing platforms to automate reminder delivery to mothers for post-partum diabetes screening when a diagnosis of GDM is recorded in their electronic health records. Multiple modalities of info-communication technology are currently available for mothers to fix their OGTT appointments, from phone-calls to mobile applications with various healthcare providers.

Lastly, we must also encourage mothers to take charge of their own health. This can be achieved through interventions to increase their health literacy and specific preventive measures against T2DM. Healthcare professionals and policy-makers can assist to elevate their self-efficacy and risk awareness via official portals of health education and organizing such programs at the healthcare facilities.

## Strength and Limitations

A key strength of this study is the novel use of the SEM to stratify the facilitators and barriers towards post-partum diabetes screening among mothers with GDM. The model facilitates the formulation of action points targeting personal, interpersonal, organizational, and public policy factors.

The results from this study reflect the perspectives of local Asian mothers who were recruited from primary care. Nevertheless, we have used the findings from this study to construct a questionnaire for a cross-sectional survey to quantify the magnitude of the individual facilitators and barriers identified. The triangulation of the results from both the qualitative study and survey will allow us to develop and prioritize multi-pronged interventions to enhance the enablers and mitigate the barriers within each SEM domain.

The qualitative research method used in this study restricts the generalizability of the findings to the general female population in Singapore. Purposive sampling was deployed to recruit women of different ethnic groups but eventually proportionately more Malay women were interviewed compared to Chinese and Indians. The recruitment was dependent on the provision of written consent.

Another potential limitation is the recruitment of the women from a single public primary care clinic. However, these women have access to both public and private primary healthcare services, so the site of recruitment is unlikely to affect their demographic profiles significantly. More tertiary educated women were interviewed, which could reflect their higher confidence and language proficiency to interact with the interviewer in English. The subsequent questionnaire survey will allow analysis of the impact of ethnicity and educational status on postnatal diabetes screening in women with GDM, which is not appropriate in a qualitative research study.

## Conclusion

Facilitators and barriers of post-partum diabetes screening for mothers with GDM are not only related to their personal and interpersonal factors but are also influenced by the local health system and policies. The multitude of socio-ecological factors must be acknowledged and addressed to improve the screening rates. Educating mothers on the benefits and risks of testing, assisting them in managing competing demands and policies promoting adherence to clinical practice guidelines across all healthcare providers may be packaged as a multi-dimensional intervention to improve the uptake of post-partum diabetes screening.

## Data Availability Statement

The raw data supporting the conclusions of this article will be made available by the authors, without undue reservation.

## Ethics Statement

The studies involving human participants were reviewed and approved by SingHealth Centralised Institution Review Board. The patients/participants provided their written informed consent to participate in this study.

## Author Contributions

SS contributed to development of the interview guide, conducted the interviews, transcribed the interviews, coded the transcribed interviews. RM, SA, and NT contributed to development of the interview guide, conception and design of the study, and revision of the manuscript. CL, YT, YS, XH, MG, LT, SL, SK, YL, and KV were involved in designing the study, creating the interview guide, coding, and organization of the themes from the data collected and drafting of the manuscript. All authors contributed to the article and approved the submitted version.

## Conflict of Interest

The authors declare that the research was conducted in the absence of any commercial or financial relationships that could be construed as a potential conflict of interest.
